# Conserved retinoblastoma protein-binding motif in human cytomegalovirus UL97 kinase minimally impacts viral replication but affects susceptibility to maribavir

**DOI:** 10.1186/1743-422X-6-9

**Published:** 2009-01-21

**Authors:** Rachel B Gill, Samuel L Frederick, Caroll B Hartline, Sunwen Chou, Mark N Prichard

**Affiliations:** 1 Department of Pediatrics, University of Alabama School of Medicine, Birmingham, AL, USA; 2 Medical and Research Services, VA Medical Center and Oregon Health and Science University, Portland, OR, USA; 3 Department of Cell Biology, University of Alabama School of Medicine, Birmingham, AL, USA

## Abstract

The UL97 kinase has been shown to phosphorylate and inactivate the retinoblastoma protein (Rb) and has three consensus Rb-binding motifs that might contribute to this activity. Recombinant viruses containing mutations in the Rb-binding motifs generally replicated well in human foreskin fibroblasts with only a slight delay in replication kinetics. Their susceptibility to the specific UL97 kinase inhibitor, maribavir, was also examined. Mutation of the amino terminal motif, which is involved in the inactivation of Rb, also renders the virus hypersensitive to the drug and suggests that the motif may play a role in its mechanism of action.

## Findings

Human cytomegalovirus (HCMV) is a ubiquitous virus that can be problematic in immunocompromised populations, including individuals with AIDS or recipients of allograft transplants. It is the most common congenital infection in the United States [1] and sequela include permanent neurological deficits, including hearing loss [2,3]. Ganciclovir (GCV), foscarnet and cidofovir (CDV) have all been approved for the treatment of HCMV infection, but each drug is associated with dose-limiting toxicities [4]. The benzimidazole L-riboside maribavir (MBV) is currently in Phase III clinical trials for the treatment of HCMV infections and inhibits viral replication by a distinct mechanism involving the direct inhibition of UL97 kinase activity [4-7]. While this drug clearly inhibits the enzymatic activity of the UL97 kinase in infected cells, the consequences of its inhibition are complex and incompletely understood as the kinase affects many cellular and viral processes.

The UL97 serine/threonine kinase is expressed early in infection and is found within the tegument of infectious virions [8,9]. Although the kinase is not required for viral replication, null mutants exhibit severe replication deficits [10], which is consistent with the inhibitory effects of MBV [5]. This enzyme has been shown to phosphorylate viral proteins including itself, ppUL44 and pp65 [11-13], as well as the large subunit of RNA polymerase II, eukaryotic elongation factor 1delta, P32 and lamins A/C [14-16]. The tumor suppressor retinoblastoma (Rb) has also been shown to be hyperphosphorylated in cells infected with HCMV [17], and this phosphorylation is dependent on UL97 kinase activity [18]. This report also showed that mutations in either the essential lysine (K355) or the conserved LxCxE Rb-binding motif in the amino terminus of pUL97 reduced the inactivation of Rb [18]. A separate study showed that the kinase phosphorylated Rb directly and did not require other proteins [19]. This activity is intriguing since Rb is also targeted by many viruses, including human papilloma virus, simian virus 40, and adenovirus [20]. However, the interaction seems to be finely-tuned between HCMV and the cell and does not appear to result in an oncogenic phenotype exhibited by other viruses which target Rb.

Rb belongs to the family of pocket proteins which prevent the progression of the cell through the G_1_/S checkpoint by binding to and suppressing the function of the transcription factor E2F; hyperphosphorylation of Rb causes it to release E2F which activates key steps in the cell cycle [21]. The inactivation of Rb by the kinase is presumed to modify cell checkpoint protein expression and induce the expression of cellular proteins required for viral infection, but its impact on viral replication has not been established. The UL97 gene product contains three consensus binding sequences for Rb [18,19]; disruption of the essential lysine or the amino terminal Rb-binding motif (LxCxE) reduces the Rb phosphorylation seen in HCMV wild type (wt) inoculated cells [18]. Therefore, we hypothesized that viruses with disrupted Rb-binding sites in the UL97 kinase might have an impaired replication phenotype or altered susceptibility to antiviral drugs.

**Figure 1:**
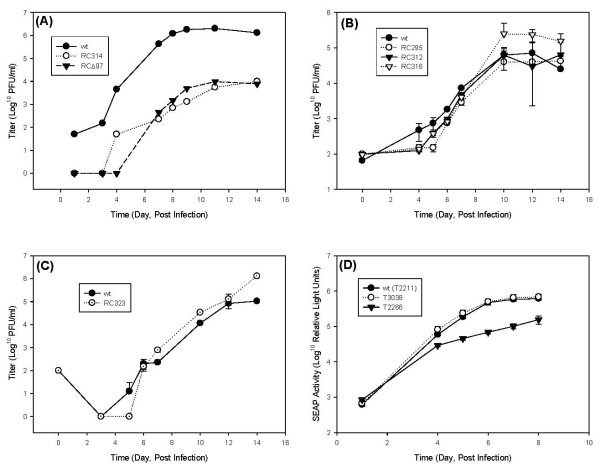
**Growth curves of HCMV recombinants with mutations in *UL97***. HFF cells were infected at an MOI of 0.01 PFU/cell and titers of the resulting progeny virus in cell lysates were determined at the indicated times. (A) Both a *UL97*-deleted virus (black triangles) and a null mutant with a K355M mutation (open circles) replicate poorly compared to the HB5 parent virus (black circles). (B) Replication kinetics of recombinant viruses with mutations in putative Rb-binding sites were determined and exhibit a slight delay in replication. Shown are the average titers from 2 replicate cultures with error bars representing the standard deviation values. RC295 (open circles) contains a mutation in the LxCxE motif, RC312 (black triangles) has a mutation in the LxCxD motif, RC316 (open triangles) carries a mutation in the IxCxE motif, and titers of the parent virus are shown as black circles. (C) RC323 (open circles) contains mutations in both the LxCxE and the LxCxD motifs and is shown with HB5 as a control (black circles). (D) Growth curves of SEAP-expressing HCMV recombinants with mutations in *UL97 *were examined separately; shown is the average SEAP activity of 5 replicates with the error bars representing the standard deviations. Replication of the wild-type virus with a SEAP-expression cassette (black circles) is similar to that of the recombinant virus with a deletion of codon 151 which disrupts the LxCxE motif (open circles), and both replicate much better than the virus with a truncation of the UL97 open reading frame (black triangles).

Recombinant viruses were engineered with point mutations in the *UL97 *open reading frame (ORF) and their construction was reported previously [18]. RC314 is a kinase-null virus with a K355M mutation. Recombinants RC295, RC312, and RC316 contain amino acid substitutions C151G (LxCxE), C428G (LxCxD) and C693G (IxCxE), respectively, that disrupt individual putative Rb-binding elements in the amino acid sequence of *UL97*. Another virus was constructed that contains both the C151G and the C428G mutations (RC323). Replication kinetics of each of these viruses were evaluated in low MOI infections of stationary HFF cells in the presence of 2% FBS. Disruption of the essential kinase motif in the *UL97 *ORF with a K355M mutation severely impacted the replication of the virus and was indistinguishable from that of RCΔ97 [Figure 1A]. This confirmed that the absence of kinase activity was responsible for the replication deficiency in the deletion mutant [10], and is consistent with results published previously [18]. Disruption of individual Rb binding motifs had minimal impact on virus replication in a 14-day time course [Figure 1B]. Growth curves for RC312, RC295, RC316, and RC323 were similar to that of the wt virus (HB5), although there appeared to be a slight delay at 4 and 5 days post infection, with RC295 having the lowest titers. This result was repeatable and was confirmed in a second independent experiment with these viruses (data not shown). The double-mutant virus (RC323) also exhibited a minor replication delay in a separate experiment [Figure 1C]. These data suggest that the mutation of these sites has a minimal effect on viral replication.

A second set of viruses was constructed that contained a secreted alkaline phosphatase (SEAP)-expression cassette at US6 (T2211) [22]. The LxCxE motif was disrupted with a C151 deletion (T3038) and pUL97 was truncated at codon 536 to yield a null mutant (T2266). Replication of these viruses was assessed in human embryonic lung (HEL) cells infected at an MOI of 0.01–0.03 PFU/cell, and SEAP supernatant activity was determined with a chemiluminescent substrate. No detectable differences were observed between the wt virus (T2211) and the UL97 ΔC151 virus (T3038) [Figure 1D]. The UL97-truncated virus (T2266) replicated poorly and had growth characteristics similar to those of RC314, and confirmed that defects in kinase activity could be detected in these studies. Results from both experiments indicated that mutation of the LxCxE motif did not severely impact virus replication. The minor delay in virus replication was not apparent in the T3038 and is likely related to assay differences. We conclude that the disruption of each of the Rb binding motifs individually or of the two amino terminal motifs in tandem has only minor effects on viral replication in cell culture. However, we cannot exclude the possibility that the disruption of all three may impact growth or that replication may be more compromised in other systems.

The mutations also had the potential to impact the efficacy of MBV and GCV, so the susceptibility of the mutants was determined by standard plaque reduction assays [23]. MBV was obtained from the National Institute of Allergy and Infectious Diseases (NIAID), CDV was a gift from Gilead Sciences and GCV was obtained from University of Alabama, Birmingham Hospital Pharmacy. All of the viruses were equally susceptible to the CDV control and was expected since this nucleotide analog does not require initial phosphorylation by the UL97 kinase [Table 1]. The kinase null virus, RC314, exhibited reduced susceptibility to GCV and MBV and is similar to results reported previously for RCΔ97 [23]. None of the viruses with mutations in the Rb binding motifs exhibited significant differences in their sensitivity to GCV, and confirmed that the mutations did not impact the enzymatic activity of the kinase. Interestingly, the disruption of the amino terminal LxCxE motif in RC295 rendered it modestly hypersensitive to MBV, which suggests that the Rb binding motif might be related to the mechanism of action of MBV. This is consistent with a previous report, which showed only this motif appeared to impact Rb phosphorylation [18]. It was not clear, however, why this mutation did not affect MBV susceptibility of the double mutant, but this effect was difficult to measure because of the assay-to-assay variability with this virus.

**Table 1:** UL97 recombinant sensitivity to Maribavir

Virus	AA Mutation	Rb-binding Motif	MBV^*a*^	CDV	GCV
HB5	wt	wt	0.37 ± 0.03	0.15 ± 0.05	3.6 ± 3
RC314	K355M	wt	14 ± 5	0.1 ± 0.07	38 ± 10
RC295	C151G	LxCxE	0.22 ± 0.08^c^	0.18 ± 0.04	2.8 ± 0.9
RC312	C428G	LxCxD	0.39 ± 0.2	0.22 ± 0.1	4.2 ± 2
RC316	C693G	IxCxE	0.3 ± 0.1	0.18 ± 0.1	3.2 ± 3
RC323	C428G/C151G	LxCxD/LxCxE	0.5 ± 0.2	0.08 ± 0.05	3.5 ± 3
T2211^*b*^	wt	wt	0.100 ± 0.008	0.23 ± 0.01	1.16 ± 0.18
T3038^*b*^	ΔC151	LxCxE	0.031 ± 0.002^c^	0.27 ± 0.01	1.03 ± 0.13

a. Values shown are the concentrations of drugs (μM) that are sufficient to reduce viral replication by 50% (EC_50_) with standard deviation shown. Each value is the average of at least 3 experiments.

b Values were obtained using a surrogate assay from viruses containing a SEAP expression cassette at US6.

c. Values were reduced significantly from the isogenic control viruses as determined by the Student's T-test (p < 0.05).

To confirm these data, the susceptibility of the SEAP-reporter viruses was also evaluated against GCV and MBV using SEAP activity as a surrogate marker of viral replication. Data were obtained by infecting confluent HEL fibroblasts at an MOI of 0.01–0.03 PFU/cell and SEAP activity in the supernatant was determined 6 days following infection [24]. MBV sensitivity data are the average of 8 determinations from 3 independent experiments for the T3038 and T2211 viruses tested simultaneously; the GCV EC_50 _values are consistent with historical data and are shown as the average of triplicate determinations. These data confirmed that the disruption of the amino terminal Rb-binding domain resulted in significantly increased sensitivity to MBV. While the EC_50 _values differ between the plaque reduction assays and the SEAP assays, both assays are consistent in that they both show that disruption of the amino terminal Rb binding motif results in increased susceptibility to MBV. These suggest that the mechanism of action of MBV may involve the LxCxE Rb binding motif and is consistent with the idea that the prevention of Rb inactivation by MBV might be an important aspect of its mechanism of action.

Disruption of the central cysteines in the UL97 Rb-binding motifs does not result in a severe replication deficiency reminiscent of kinase null viruses, but rather results in a very modest delay in viral replication. This might suggest that the inactivation of Rb is not a crucial function in HFF or HEL cells and that it may be important only in vivo. Alternatively, it is possible that the motifs are redundant and all of them must be deleted to impact replicative ability of the virus. Nevertheless, the C151G mutation in the RC295 virus and the C151 deletion in the T3038 virus clearly conferred hypersensitivity to MBV when compared to their isogenic controls. While potential mechanisms for this are unclear, it indicates that mechanism of action of the drug is related to the function of the amino terminal Rb-binding motif at some level. Additional experiments will be required to assess the potential impact of double and triple mutants. Nonetheless, these findings offer insight into a new aspect of MBV activity and additional studies with the inhibitor together with genetic studies will help define the function of the kinase in viral infection.

## Abbreviations

Rb: retinoblastoma protein; HCMV: human cytomegalovirus; GCV: ganciclovir; CDV: cidofovir; MBV: maribavir; SEAP: secreted alkaline phosphatise; MOI: multiplicity of infection; HEL: human embryonic lung fibroblasts; NIAID: National Institute of Allergy and Infectious Diseases; ORF: Open reading frame; HFF: human foreskin fibroblasts; wt: wild type.

## Competing interests

The authors declare that they have no competing interests.

## Authors' contributions

RBG participated in virus production and data analysis and drafted the manuscript. SLF carried out virus production. CLH performed drug sensitivity studies and growth curve analyses. SC participated in construction and analyses of SEAP virus experiments. MNP participated in experimental design and implementation and assisted in drafting the manuscript. All authors read and approved the final manuscript.
